# The Therapeutic and Pathogenic Role of Autophagy in Autoimmune Diseases

**DOI:** 10.3389/fimmu.2018.01512

**Published:** 2018-07-31

**Authors:** Heng Yin, Haijing Wu, Yongjian Chen, Jianzhong Zhang, Min Zheng, Genhui Chen, Linfeng Li, Qianjin Lu

**Affiliations:** ^1^Department of Dermatology, Hunan Key Laboratory of Medical Epigenomics, The Second Xiangya Hospital, Central South University, Changsha, China; ^2^Department of Dermatology, Peking University People’s Hospital, Beijing, China; ^3^Department of Dermatology, The Second Affiliated Hospital, Zhejiang University School of Medicine, Hangzhou, China; ^4^Beijing Wenfeng Tianji Pharmaceuticals Ltd., Beijing, China; ^5^Department of Dermatology, Beijing Friendship Hospital, Capital Medical University, Beijing, China

**Keywords:** autophagy, light-chain 3, Agt, autoimmunity, LAP

## Abstract

Autophagy is a complicated cellular mechanism that maintains cellular and tissue homeostasis and integrity *via* degradation of senescent, defective subcellular organelles, infectious agents, and misfolded proteins. Accumulating evidence has shown that autophagy is involved in numerous immune processes, such as removal of intracellular bacteria, cytokine production, autoantigen presentation, and survival of lymphocytes, indicating an apparent and important role in innate and adaptive immune responses. Indeed, in genome-wide association studies, autophagy-related gene polymorphisms have been suggested to be associated with the pathogenesis of several autoimmune and inflammatory disorders, such as systemic lupus erythematosus, psoriasis, rheumatoid arthritis, inflammatory bowel disease, and multiple sclerosis. In addition, conditional knockdown of autophagy-related genes in mice displayed therapeutic effects on several autoimmune disease models by reducing levels of inflammatory cytokines and autoreactive immune cells. However, the inhibition of autophagy accelerates the progress of some inflammatory and autoimmune diseases *via* promotion of inflammatory cytokine production. Therefore, this review will summarize the current knowledge of autophagy in immune regulation and discuss the therapeutic and pathogenic role of autophagy in autoimmune diseases to broaden our understanding of the etiopathogenesis of autoimmune diseases and shed light on autophagy-mediated therapies.

## Introduction

**Figure 1:**
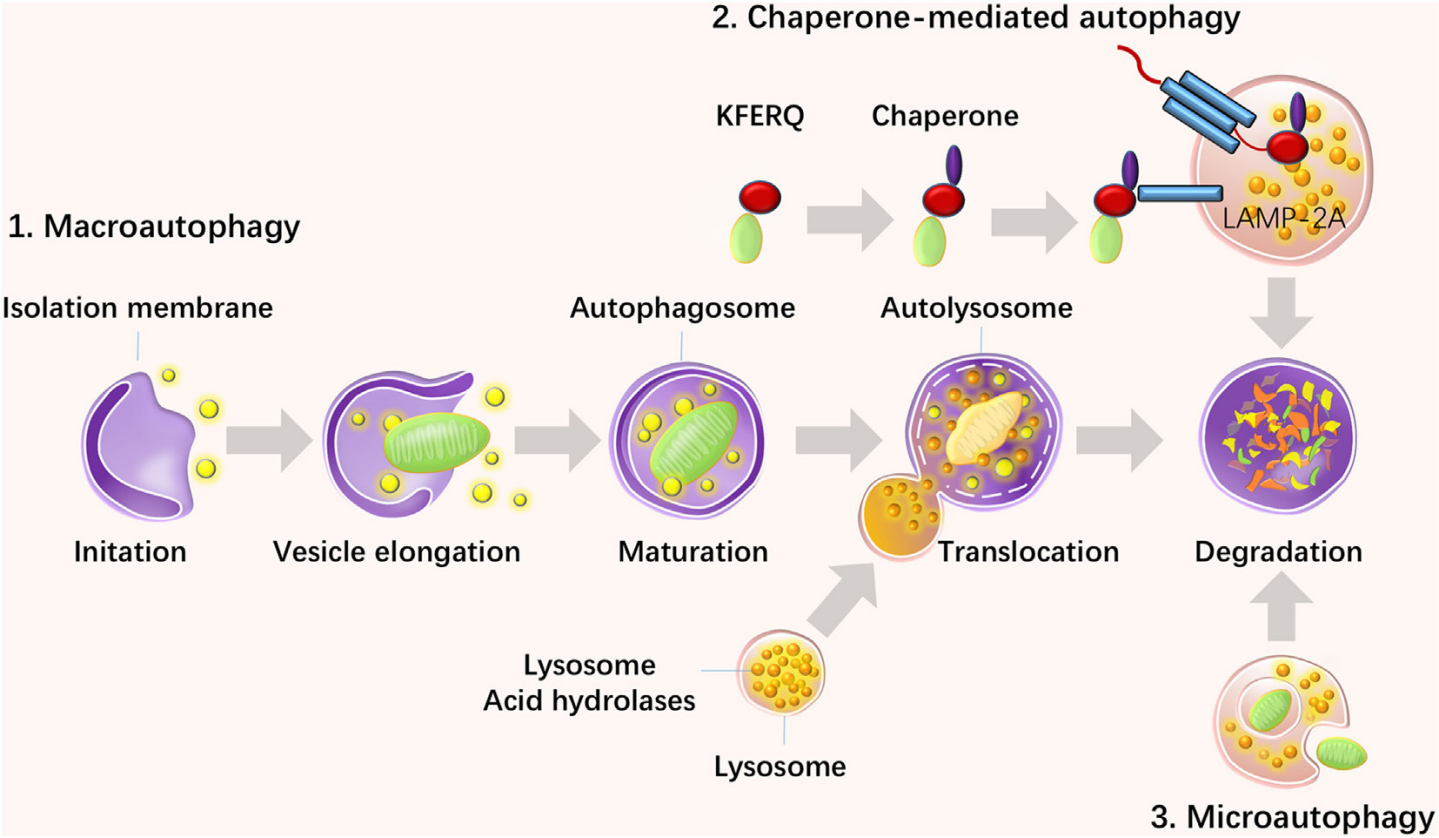
Three types of autophagy and their steps. There are three types of autophagy: macroautophagy, chaperone-mediated autophagy (CMA), and microautophagy. Macroautophagy initiates with the recruitment of protein aggregates and misfolded proteins by phagophores. Then, vesicles undergo elongation and form double-membraned vesicles, called autophagosomes, and the cytoplasmic components are enclosed *via* cargo sequestration and fused with lysosomes for degradation and recycling. CMA is another type of autophagy, which is involved in the direct recognition, targeting, and degradation of substrates by lysosomes rather than autophagosome formation. Microautophagy is a process of lysosomal engulfment of cytoplasmic cargo with the formation of autophagic tubes and vesicles.

Autophagy was discovered more than 40 years ago and has recently become a topic of increasing interest since the Japanese biologist Yoshinori Ohsumi won the Nobel Prize in 2016 for the discovery of this “self-eating” mechanism. Autophagy refers to a survival mechanism that cells use to degrade unwanted and useless organelles, proteins, and infectious agents to maintain homeostasis (1). There are three types of autophagy: macroautophagy, chaperone-mediated autophagy (CMA), and microautophagy. Among them, macroautophagy is the most intensively studied and is referred to as autophagy in general, and is the focus of this review. Macroautophagy occurs in all eukaryotic cells and initiates the recruitment of protein aggregates and misfolded proteins by phagophores. Then, vesicles undergo elongation and form double-membraned vesicles, called autophagosomes, and then the cytoplasmic components are enclosed *via* cargo sequestration and fuse with lysosomes for degradation and recycling (2) (Figure 1). Macroautophagy is capable of constitutively delivering cytosolic proteins for MHC-II presentation. Autophagosomes are capable of fusing with multi-vesicular bodies or enodosomes and MHC-II loading compartments (3). CMA is another type of autophagy, which is involved with the direct recognition, targeting, and degradation of substrates by lysosomes, rather than by autophagosome formation (4). As a substrate of CMA, a protein should contain the amino acid sequence of the polypeptide motif KRERQ (5). Target proteins are selectively recognized by cytosolic heat shock cognate 70/co-chaperones and then delivered to the lysosomal membrane. Proteins then bind to the integral lysosomal membrane protein (LAMP-2A) and unfold and reach the lumen *via* a LAMP-2A-enriched translocation complex. Then proteins undergo degradation in the autolysosome (6). In addition, microautophagy is the process of lysosomal engulfment of cytoplasmic cargo with the formation of autophagic tubes and vesicles (7). Cytosolic and soluble proteins and particulate cellular constituents are directly internalized in single-membrane vesicles into lysosome by invaginating, protrusion, and or septation of the lysosomal limiting membrane (8). Microautophagy is a process of detect invagination and fusion of the vacuolar/lysosomal membrane under the limited nutrient status. Therefore, microautophagy is critical for cell survival, particularly for cells that are under stress such as nutrient starvation (9).

It is now widely accepted that macroautophagy (autophagy for short) is involved in several pathophysiological processes and complex diseases, such as autoimmune disorders, cancer, and metabolic disorders. Indeed, autophagy has been found to play four principal roles in immune responses: intracellular pathogen removal, lymphocyte development, pro-inflammatory signaling, and the secretory pathway (10–12). The combined data from genome-wide association studies (GWAS) and inhibition assays in mouse models have implicated autophagy in autoimmune diseases, especially in systemic lupus erythematosus (SLE), psoriasis, rheumatoid arthritis (RA), inflammatory bowel disease (IBD), and multiple sclerosis (MS). Therefore, this review will summarize the current understanding of the molecular regulation of autophagy and its roles in immunity and discuss the therapeutic and pathogenic role of autophagy in these autoimmune disorders, providing potential diagnostic targets and therapeutic strategies for autoimmune diseases. Although CMA and microautophagy has been found to be also related with immune responses (13), the regulations from these two pathways will not be discussed in this review.

## Molecular Regulation of Autophagy

**Figure 2:**
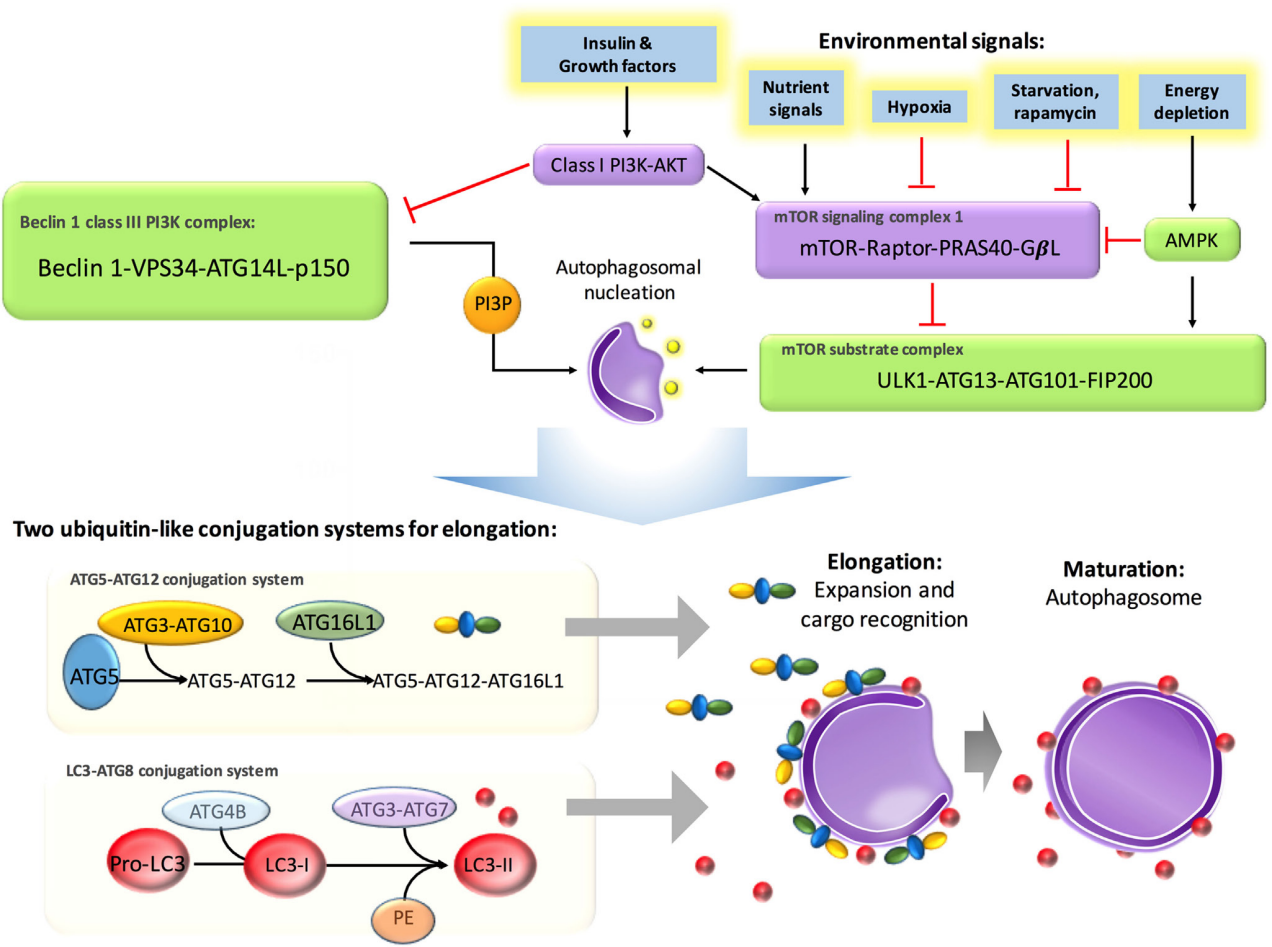
Molecular regulation in autophagy. Autophagy is suppressed by insulin and other growth factor signaling, starvation, and nutrient signals but activated by energy depletion and AMBRAs, BIF1, and UVRAG. Energy depletion can activate AMP-activated protein kinase (AMPK) and further activate the mammalian target of rapamycin (mTOR) substrate complex, which consists of phosphorylated UNC-51-like kinase 1 (ULK1), ATG13, ATG101, and FIP200. In addition, this pathway positively regulates the formation of autophagosomes. Environmental signals, such as starvation, repress autophagy *via* inhibition of mTOR, which is located in the mTOR signaling complex 1, formed by the regulatory-associated protein of mTOR (Raptor), G protein beta subunit-like protein (GβL), and proline-rich Akt/PKB substrate 40 kDa (PRAS40). Insulin and other growth factor signaling activates class I PI3K–Akt, which inhibits autophagy *via* activation of mTOR signaling complex 1 and inhibition of the Beclin 1 class III PI3K complex, which contains Beclin 1, class III phosphatidylinositol-3-kinase (PIK3C3), and ATG14L. Autophagosomal elongation requires two ubiquitin-like conjugation systems: the ATG5–ATG12 conjugation system and light-chain 3–ATG8 conjugation system.

It has been well established that autophagy is predominately regulated by the autophagy-related gene (*Atg*) family, which initiates the formation of autophagosomes (14). Energy depletion can activate AMP-activated protein kinase and further activate the mammalian target of rapamycin (mTOR) substrate complex, which consists of phosphorylated UNC-51-like kinase 1 (ULK1), ATG13, ATG101, and FIP200. In addition, this pathway positively regulates the formation of autophagosomes (15). Environmental signals, such as starvation, repress autophagy *via* inhibition of mTOR, which is located in the mTOR signaling complex 1, formed by the regulatory-associated protein of mTOR (Raptor), G protein beta subunit-like protein, and proline-rich Akt/PKB substrate 40 kDa (16). In addition, insulin and other growth factor signaling activates Class I PI3K–Akt, which inhibits autophagy *via* activation of the mTOR signaling complex 1 and inhibition of the Beclin 1 class III PI3K complex, which contains Beclin 1, class III phosphatidylinositol-3-kinase (PIK3C3), and ATG14L (17). It has been documented that autophagosomal elongation requires two ubiquitin-like conjugation systems: the ATG5–ATG12 conjugation system and light-chain 3 (LC3)–ATG8 conjugation system (16). When cells take up phagocytosed dead cells, LC3-associated phagocytosis (LAP) will take place to promote digestion (18). The molecular regulation of autophagy has been summarized in Figure 2.

## Autophagy-Mediated Regulation in Immunity

It has been reported that autophagy is initiated by different families of pathogen-recognition receptors [such as toll-like receptors (TLRs)], damage-associated molecular patterns (such as HMGB1 and misfolded proteins), pathogen receptors, IFN-gamma, DAP kinase, JNK, CD40, TNF-alpha, and NF-κB (19). And it is repressed by Th2 cytokines, Bcl-2, and canonical nutrient sensing insulin–AKT–TOR pathway. Therefore, it is no surprise that autophagy involves in innate and adaptive immune response.

### Removal of Pathogens by Autophagy

Autophagy participates in innate immunity *via* removing intracellular microbial pathogens and protecting the cytosol. There are two major ways to remove intracellular pathogens by autophagy in dendritic cells (DCs), macrophages, and other phagocytes. One is called xenophagy, which eliminates pathogens by engulfing them in double-membrane autophagosomes. This process is activated during infection by TLRs (20). Many bacteria and parasites can be removed by xenophagy. The other way is mediated by microtubule-associated protein LC3 and named LAP, which encloses pathogens in single-membrane phagosomes and involves LC3 (18). LAP has been found to be activated by TLR agonists and immune complexes (21), which are abundant in SLE. LAP is triggered by bacteria *via* surface markers (TLRs, FcγR, NOD, and SLAM) expressed by host cells, or by cytosolic pathogen sensing signals *via* direct induction or binding to autophagy component proteins. LAP shows capacity in dead cell clearance, which requires PtdSer receptor TIM4 to induce the recruitment of LC3 to the phagosome (22).

However, several pathogens have been found to have some strategies to avoid autophagy process. *Listeria monocytogenes* and *Shigella flexneri* have been found to express several proteins which are modified to inhibit recognition by the autophagic machinery. Human immunodeficiency virus 1 (HIV-1) can express Nef protein to interact with Beclin-1, thereby blocking the fusion of autophagosomes with lysosomes (23). This is the reason why we can be infected with bacteria and viruses even though we have normal innate immune responses.

In physiological conditions, apoptotic cells, with ATG and Bclin-1expression, expresses phosphatidylserine on cell surfaces, which is referred to as “eat-me” signals. Then they release lysophosphatidylcoline, which is considered as “come-get-me” signals. Then, these apoptotic cells can be efficiently cleared by phagocytes. However, in ATG5 or Beclin-1-deficient cells, apoptotic cells cannot express “eat-me” and “come-get-me” signals, which will lead to impaired clearance of apoptotic bodies, thereby contributing to autoimmune disorders (24).

### Antigen Processing for MHC Presentation by Autophagy

Antigen-presenting cells (APCs), including DCs, macrophages, and B cells, are the bridges between innate and adaptive immunity. MHC-I molecules present endogenous antigens to CD8^+^ T cells *via* processing them by proteasome and translocating them into ER. Whereas MHC-II presents extracellular antigens on lysosome-derived organelles to CD4^+^ T cells. Before presentation with MHC-II by APCs, extracellular pathogens need to be properly digested and processed by APCs *via* degradation pathways, which include the ubiquitin–proteasome system and autophagy. MHC-II molecules can also present intracellular antigens, such as cytosolic or nuclear antigens, *via* the fusion of autophagosomes with MHC-II rather than the lysosome (8). In addition, the process of MHC-I presentation can be enhanced by autophagy (25). This urgent MHC-I presentation which is promoted by autophagy seems to need more time and more antigens, which means that this alternative process in the “cellular emergency” situation occurs when the classical pathway is imparied.

### Lymphocyte Development, Activation, and Polarization Regulated by Autophagy

It has been reported that autophagy plays a critical role for the thymic selection, T cell development, survival, and proliferation. Atg5^−/−^ mice show reduced thymocyts and peripheral lymphocytes and increased cell death in CD8^+^ T cells. And these Atg5^−/−^ T cells fail to proliferate under TCR stimulation (26). Atg7^−/−^ T cells show deficiency in cell survival, with the expanded ER content and mitochondria (27). The similar results have been observed in the Atg3^−/−^ mice, which display impaired autophagy (28). On the other hand, some studies have claimed that autophagy promote the cell death during virus infection. For example, HIV Env-mediated autophagy induces apoptosis of CD4^+^ T cells *via* CXCR4 (29), and autophagy is involved in RIPK1-dependent necroptotic cell death when Fas-associated death domain activity and caspase-8 is insufficient (30). In addition, Beclin-1^−/−^ CD4^+^ T cells, which are deficiency in autophagy, show preference in apoptosis under the TCR stimulation. Accumulation of pro-caspase-3, pro-caspase-3, and Bim might be one of the mechanisms (31). Therefore, the role of autophagy in T cell survival remains unclear. Future studies are needed to address this issue.

There are two subsets of conventional DCs (cDCs): CD8α (CD103^+^) cDCs and CD4^+^ (CD11b^+^) cDCs. CD8α cDCs efficiently cross-present exogenous antigens on MHC-I to CD8^+^ T cells, whereas CD4^+^ cDCs more efficiently polarize CD4^+^ T cells into Th1, Th2, Th17, or regulatory T cells (Tregs) by MHC-II-restricted presentation (32). Autophagy has been reported to be essential for the CD4^+^ T-cell response by DCs (33, 34). Indeed, a deficiency of autophagy in DCs results in a mild EAE phenotype in mice (35), which is a Th17-mediated mouse model. Furthermore, autophagy has also been linked with DC-derived cytokine production, such as IL-6 and IL-12p40 (36), which is critical for T-cell activation and polarization. This evidence indicates autophagy might involve in the Th cell differentiation. Besides, Tregs have been reported to be regulated by autophagy. For example, during chronic hepatitis B virus infection, HMGB-1-induced autophagy maintains Treg cell functions (37). And *Agt16l1*gene has been found to differentially regulate Treg and Th2 cell and further control intestinal inflammation (38). However, autophagy can be also regulated by Treg cells. For example, Foxp3^+^ Treg cells have been found to suppress immune response *via* inhibiting autophagic machinery in DCs depending on CTLA4. The binding of CTLA-4 promotes activation of PI3K/Akt/mTOR axis and FoxO1 nuclear exclusion in DCs, resulting in reduced expression of autophagy component microtubule-associated protein 1 light chan 3 beta (39).

In addition, autophagy also regulates B cell survival and development. ATG5 has been identified to be critical for B-cell survival and subset maintenance, such as pre-B and mature B1a B cells (40). Autophagy has been found to be required for immunoglobulin production by plasma cells (41). Indeed, the degradation of misfolded protein is particularly important for antibody-secreting cells, in which protein synthesis and degradation must be balanced. It has been observed that increased autophagosome formation and degradation occur in activated mouse B cells during plasma cell differentiation (41), as well as in human B cells activated by CpG (42). Genetic studies have identified several autophagy-related genes essential for antibody responses and plasma cell homeostasis (43), and dysregulated autophagy contributes to the plasma cell pathology in antibody-mediated autoimmune diseases such as SLE (44, 45). In addition, autophagy has been found to contribute to IL-17-dependent plasma differentiation *via* regulating Blimp-1 expression and Beclin-1/p62-associated B cell apoptosis (46, 47). These findings show that autophagy in B cells might play a pathogenic role in antibody-mediated autoimmune disease, such as lupus.

### Pro-Inflammatory Signaling Regulated by Autophagy

Increasing evidence has demonstrated the interplay between autophagy and the NF-κB signaling pathway (48). The NF-κB family of transcription factors regulates transcription of a broad range of genes, which are engaged in cell proliferation, survival, differentiation, and development. These transcription factors are also essential in inflammation and immune responses (49). The mammalian NF-κB family contains five members: RelA, c-Rel, Rel-B, p50, and p52 (50), while inhibitors of the NF-κB protein family consist of IκBα, IκBβ, IκBε, and the IκB-like inhibitors p100 and p105 (51). It has been well documented that activation of the inhibitor of NF-κB (IκBα) kinase complex is required for the induction of autophagy. Conversely, in an Atg5- and Atg7-deficient system, autophagy has been proven to be critical for the activation of NF-κB (52). In addition, the cross talk between NF-κB and autophagy has been observed in immune cells. The regulation of T-cell receptor-mediated NF-κB activation by B-cell lymphoma/leukemia 10 is associated with autophagy adaptor p62/SQSTM1 (53), which is also found to be a modulator of NLRP3-inflammasome activation and IL-1 beta production in macrophages (54). However, in tissue-specific macrophages, autophagy has been revealed to promote NF-κB activation to boost the antifungal immune response (55). This evidence indicates a pathogenic or therapeutic role of autophagy depending on different microenvironments and signals.

### Interplay Between Cytokine Secretion and Autophagy

**Figure 3:**
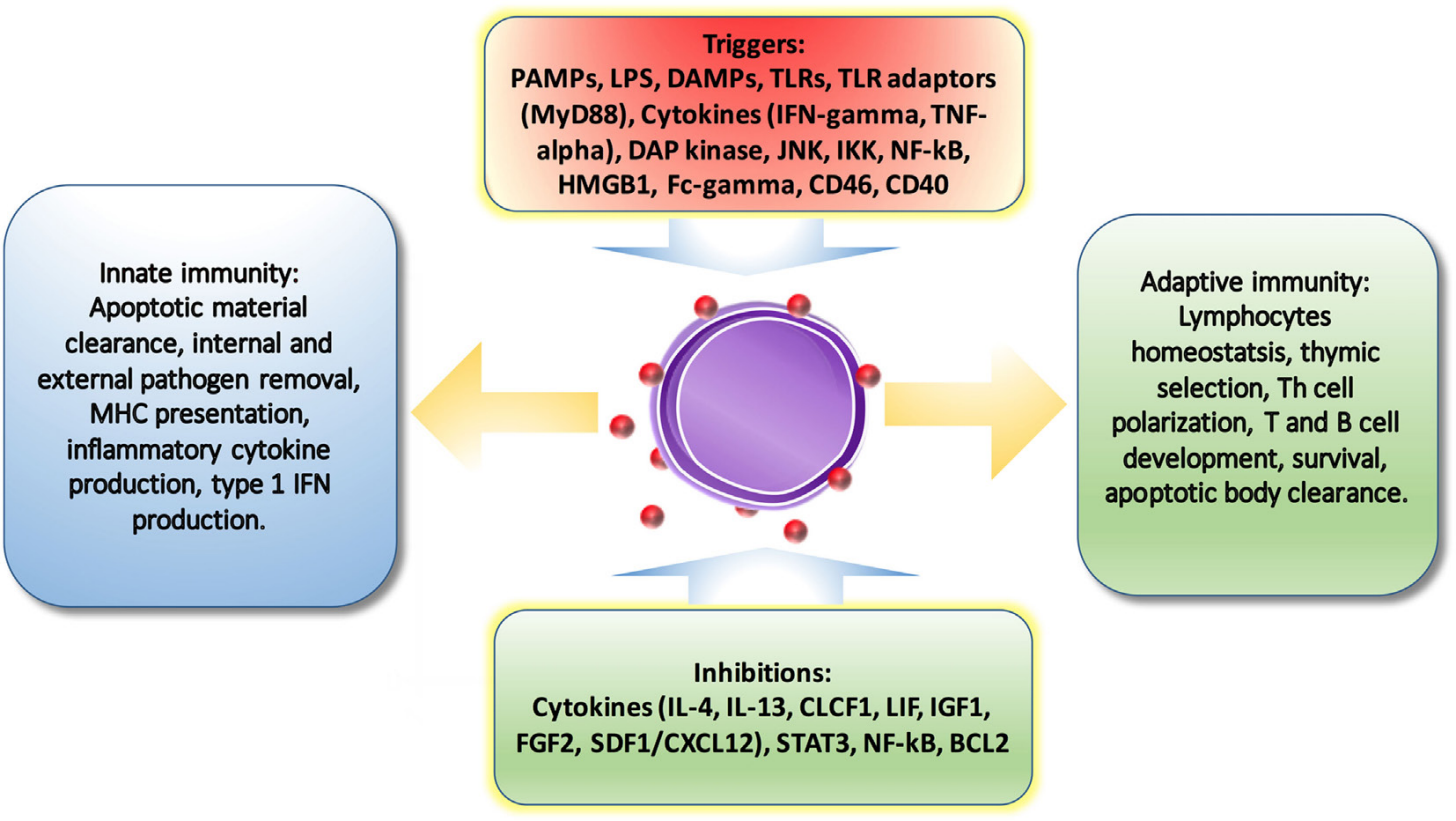
The regulations of autophagy on immune system. Autophagy is triggered and inhibited by cytokines and molecules from immune system. And also, autophagy is involved in pathogen removal, cytokine secretion, lymphocyte survival and differentiation, MHC presentation, apoptotic cell clearance, and pro-inflammatory signaling.

It is no surprise that autophagy-regulated cytokine secretion by the secretory pathway shares some common functions with phagocytosis, such as vesicle trafficking and membrane fusion, which facilitates the important role of autophagy in immune regulation. ATG5 deficiency, for example, results in elevated IL-1 alpha secretion by macrophages (56), while inhibition of autophagy leads to promotion of IL-1 beta *via* reducing degradation (57) by APCs and increases IL-23 secretion as a consequence (58), which can further promote Th17-mediated inflammatory responses. On the other hand, cytokines can also regulate autophagy. IL-10, which is an anti-inflammatory cytokine, has been found to inhibit autophagy in murine macrophages *via* activation of mTOR complex 1 (59). Another example is IL-6, which is a universal inflammatory cytokine involved in many autoimmune and inflammatory diseases. IL-6 has been illustrated to inhibit starvation-induced (60) and IFN-gamma-induced autophagy (61) by regulating Bcl-2 and Beclin1. However, IL-6 has also been found to be required for autophagy by promoting autophagosomal maturation (62, 63). The functions of autophagy have been summarized in Figure 3. Further studies are necessary to validate the interplay between autophagy and cytokines.

## The Therapeutic and Pathogenic Role of Autophagy in Autoimmune Diseases

Based on its functions in the immune system, autophagy might display a pathogenic and/or therapeutic role in autoimmune diseases, depending on the pathogenesis of the disease and the key players in disease development. The detailed findings for each autoimmune disease are elaborated upon in the following paragraphs.

### Autophagy in SLE

Systemic lupus erythematosus is a typical autoimmune disease, which is characterized by abnormal APCs and T and B cells, with abundant autoantibodies (64–66). Dysregulated production of IL-17 (67) and abnormally differentiated follicular helper T (Tfh) cells (68, 69), aberrant DCs, and plasma B cells (70) have been identified to play an essential role in the pathogenesis of SLE. Although autophagy negatively regulates IL-17 production, the inhibition of autophagy in a lupus mouse model reduced the disease phenotype by partially suppressing plasma cell differentiation and antibody production (44). In addition, GWAS in lupus cohorts have identified that several SNPs in the *Atg5* gene confer genetic susceptibility to lupus (71, 72). In addition, in a follow-up study, a SNP in the *Atg5* gene, rs573775, was identified to be related to IL-10 production and higher risk of lupus (73). In APCs, LAP has been found to be required for the trafficking of immune complexes and TLR9 into the interferon signaling pathway and to promote type 1 interferon production (21, 74), which is a key player in lupus pathogenesis. In addition, autophagy has been reported to deliver viral ligands to TLR7 in plasmacytoid DCs during vesicular stomatitis virus and Sendai virus infection and contribute to type 1 interferon production (75), which might be associated with lupus. However, defects in LAP, rather than canonical autophagy, can cause SLE-like phenotypes (76) with IL-10 production (77). Increased autophagy has been observed in T and B cells from a lupus mouse model, as well as in PBMCs from patients with SLE (44). In addition, autophagy-related genes, including mTOR, Beclin-1, LC3, and p62, have been found to be expressed differentially by lupus PBMCs (45). Blockade of macrophage autophagy ameliorates activated lymphocyte-derived DNA-induced murine lupus possibly *via* inhibition of proinflammatory cytokine production, such as IL-6 and TNF-alpha (78). Recently, IL-21, which is a key cytokine produced by Tfh cells, has been found to induce mTOR activation and further eliminate autophagy and differentiation of Treg cells (79). To summarize, ATG5 deficiency and mTOR elevation in innate immunity leads to insufficient autophagy, which results in reduced dead cells clearance, enhanced levels of cytoplasmic nucleic acid and autoantigens. As a consequence, increased type 1 IFN by DCs can induce B cell hyper-differentiation and antibody production. In adaptive immunity, high LC3 and accumulation of autophagic vacuoles can increase autophagy and promote the survival of T and B cells (8).

In addition, environmental factors, such as UV light and Epstein–Barr virus (EBV) infection, which have been shown to contribute to the initiation of lupus, have been linked to autophagy. For example, UV-induced DNA damage has been found to result in decreased expression of AMBRA1 and ULK1, which are important mechanisms in autophagy (80). In addition, autophagy has been reported to be involved in MHC-II presentation of EB nuclear antigen 1 to T cells (81) and to participate in EBV infections (82). Furthermore, severe vitamin D deficiency affected the expression of ATGs in PBMCs and T-cell subsets in active SLE patients, indicating that vitamin D may affect T-cell subsets *via* regulating autophagy (83).

In SLE treatment, autophagy has been reported to be a therapeutic target. Rapamycin is a FDA approved immunosuppressive agent for organ transplantation. It excuses its inhibitory effects on T cells *via* blocking mTORC1 (84), which is also the key player of autophagy. Rapamycin has been shown to be efficient in treating lupus mice and patients, with the decreased levels of autoantibodies, proteinuria, and prolonged survival in mice and patients (85, 86). In an off-label clinical study, refractory SLE patients were treated with rapamycin. Compared with standard treatment, the rapamycin-treated group showed decreased disease activity and prednisone requirement (87). And this suppressive effect might be conducted through inhibition on HRES 1/Rab4 and Rab5A and limiting the production of type I IFN by DCs (88). Besides, other treatments also have effects on autophagy. In a clinical observation, hydroxychloroquine, which is the most common treatment for SLE, has been found to inhibit autophagy, particularly LAP-mediated autophagy (89). Glucocorticoids can induce autophagy *via* inhibiting IP3-mediated calcium signaling and mTOR (90). Anti-CD20 mAbs can trigger autophagy by caspase-independent cell death induction (91). And anti-TNF alpha mAbs can inhibit autophagy by limiting proautophagic cytokine production (92). In a recent study, inhibition of Treg cell differentiation and IL-21 has been found to repress rapamycin axis *via* suppression of autophagy in lupus patients (79). Although different drugs have various effects on autophagy in lupus, and the role of autophagy in lupus can be friend or foe, the balance between innate immunity and adaptive immune response should be considered when consider autophagy as therapeutic target.

### Autophagy in Psoriasis/Psoriatic Arthritis

Psoriasis is an inflammatory autoimmune skin disease, which can also affect other organs. Approximately 6–42% of psoriasis patients will develop psoriatic arthritis (93). The pathogenesis of psoriasis is unclear. However, increased epithelial keratinocyte proliferation is an essential characteristic of psoriasis (94), and IL-17 and other inflammatory cytokines have been revealed to play an important role in the development of psoriasis. There is no direct evidence to show whether ATG16L1 contributes to psoriasis. However, defects in autophagy have been found to result in proinflammatory cytokine production and keratinocyte proliferation *via* increased p62 expression (95). Enhanced expression of the autophagy-related gene *SQSTM1* has been observed in psoriatic skin lesions (95). In addition, mutation of the psoriasis risk gene *AP1S3* has been found to result in impaired autophagy and increased skin inflammation (96), and increased expression of ATG16L1 has been observed in DCs from psoriatic arthritic patients (97). The inhibition of autophagy *via* activation of PI3K/AKT/mTOR has been suggested as a therapeutic method for the treatment of IL-17a-mediated psoriasis (98). In addition, other studies have shown the therapeutic role of autophagy in psoriasis *via* inhibition of IL-17a production (58). In addition, the inhibition of autophagy by chloroquine may accelerate psoriasis *via* promoting IL-23 production (99). In addition, vitamin D, sirolimus, retinoids, and UVB therapy, which can promote psoriasis, can induce the activation of autophagy (100–103). Taken together, these findings indicate that autophagy shows a therapeutic role in this disease.

### Autophagy in MS

Multiple sclerosis is an inflammatory disorder that is characterized by immune system reactivity against myelin in the central nervous system, resulting in varying degrees of either relapsing or progressive neurological degeneration. ATG5 (104) and immune-related GTPase M (IRGM) 1 are increased, while ATG16L2 is decreased, in autoreactive T cells in EAE and actively relapsing-remitting MS brains (105). Inhibition of autophagy by conditional knockout of Beclin-1 in CD4^+^ T cells has shown a protective role in the EAE model (31). Similar effects have been observed in an ATG7 conditional knockout system (35). Administration of rapamycin reduces relapsing–remitting EAE *via* inhibition of autophagy (106), indicating a pathological role of autophagy in MS.

### Autophagy in RA

Rheumatoid arthritis is a chronic and systemic inflammatory autoimmune condition that primarily affects the joints and is characterized by progressive destruction of the joints. The pathogenesis of RA remains unclear. However, dysregulated immune cells, such as Th17 cells, Tfh cells, macrophages, B cells, and fibroblast-like synoviocytes have been identified to contribute to this disorder (107). Fibroblasts are a key player, and their survival has been found to be regulated by autophagy induction and CHOP underexpression under endoplasmic reticulum stress (108). Increased levels of autophagy have been observed in the synovial tissues from patients with active RA and are correlated with disease activity (109). However, the effect of autophagy on the survival of RA synovial fibroblasts is controversial (110). In another study, reduced expression of ALFY and the formation of p62-positive polyubiquitinated protein aggregates promote cell death in RA synovial fibroblasts under severe ER stress (111). Moreover, IL-17-mediated mitochondrial dysfunction has been found to impair apoptosis in RA synovial fibroblasts through activation of autophagy (112).

In immune cells, increased autophagy has been observed in RA CD4^+^ T cells, resulting in T-cell hyperactivation and resistance to apoptosis (113). The inhibition of autophagy *via* an ATG7 knockdown system showed impaired bone destruction in TNF-mediated arthritis (114), partially resulting from the reduced production of IL-6 and IL-1 by inhibition of autophagy. The autophagy-related protein optineurin has also been found to negatively regulate osteoclastogenesis by modulating NF-κB and IFN-β signaling (115). Taken together, these findings indicate that autophagy shows a pathological role in RA by regulating inflammatory cytokines and bone destruction.

### Autophagy in IBD

Inflammatory bowel disease refers to two different chronic conditions or diseases that may be related, Crohn’s disease and ulcerative colitis, which consist of inflammation of the wall of the bowel or intestines. GWAS have identified ATG16L1 and immunity-related IRGM in Crohn’s disease (116), indicating a role of autophagy in the pathogenesis of IBD. Altered expression of IRGM leads to Crohn’s disease with defective autophagy (117). In addition, IBD is an IL-17a- and IL-1 beta-mediated disease. The deletion of ATG16L1 also leads to increased IL-1 beta production in macrophages (118), which might contribute to IBD. Moreover, ATG16L1 and nucleotide-binding oligomerization domain-containing protein 2 interact in an autophagy-dependent antibacterial pathway implicated in Crohn’s disease pathogenesis (119).

## Conclusion

The interplay between autophagy and the immune system emphasizes an important role of autophagy in the pathogenesis of autoimmune diseases. Autophagy is involved in pathogen removal, cytokine secretion, lymphocyte survival and differentiation, MHC presentation, apoptotic cell clearance, and pro-inflammatory signaling. In both *in vivo* and *in vitro* systems, inhibition of autophagy ameliorates diseases including SLE, MS, and RA. However, in other cases, it seems to exacerbate diseases such as psoriasis, psoriatic arthritis, and IBD. Even in RA, autophagy shows a therapeutic and pathogenesis role in the survival of RAFLS (Table 1). In addition, the regulation of autophagy varies in different tissues and cells. This evidence means that extreme care should be exercised if autophagy is to be utilized as a therapeutic target. Individual differences, even in the same types of diseases, should be considered. For example, SLE is a heterogeneous disease, and lupus patients might be either predominated by IL-17a and/or IL-21 expression. The outcome might be totally different if autophagy inhibition is applied in these two different types of patients. Therefore, further investigation is needed to clarify the regulation of autophagy in each autoimmune disease, and personalized therapy is strongly recommended in the future.

**Table 1:** The regulation of autophagy in autoimmune diseases.

Diseases	Cell types	Autophagy related genes and proteins	Effects	Reference
Systemic lupus erythematosus (SLE)	White blood cells	*Atg5*	Regulating IL-10 production	(71–73)
SLE	Plasma cells	*Atg7*	Regulating plasma cell differentiation	(43)
SLE	Antigen-presenting cells (APCs)	LAP	Regulating interferon and IL-6, TNF-alpha, and IL-10 production	(21, 74–77)
SLE	PBMCs	Mammalian target of rapamycin (mTOR), Becline-1, light-chain 3 and p62	Expressed differentially	(45)
SLE	APCs	AMBRA1 and UNC-51-like kinase 1 (ULK1)	UBV induced lower expression of AMBRA1 and ULK1	(80)
SLE	APCs and T cells	–	Involving in the Epstein–Barr virus infections	(81, 82)
SLE	PBMCs and T cells	–	Vitamin D affects T-cell subsets *via* regulating autophagy	(83)
Psoriasis	Keratinocytes	ATG16L1 *SQSTM1*	Regulating keratinocytes proliferation	(95)
Psoriatic arthritis	Dendritic cells	ATG16L1	–	(97)
Psoriasis	T cells	PI3K/AKT/mTOR	Regulating IL-17 production	(98)
Multiple sclerosis (MS)	T cells	ATG5, immune-related GTPase M (IRGM)1, ATG16L2	Expressed differentially	(104, 105)
MS	T cells	Beclin-1, ATG7, mTOR	Knockdown or inhibition shows protective role in EAE	(31, 35, 106)
Rheumatoid arthritis (RA)	RA synovial fibroblasts	ALFY, p62	Regulating survival of RA synovial fibroblasts	(111, 112)
RA	RA synovial fibroblasts	–	Impairing apoptosis	(113)
RA	T cells	ATG7, optineurin	Regulating pro-inflammatory cytokine production	(114, 115)
Inflammatory bowel disease (IBD)	–	ATG16L1, IRGM	SNPs, IL-17a, and IL-1beta production	(116, 117)
IBD	Macrophages	ATG16L1, nucleotide-binding oligomerization domain-containing protein 2	Regulating IL-1beta production	(118, 119)

## Author Contributions

HY and HW wrote the manuscript. YC, JZ, MZ, LL, and GC edited the manuscript. QL revised the manuscript.

## Conflict of Interest Statement

The authors declare that the research was conducted in the absence of any commercial or financial relationships that could be construed as a potential conflict of interest. The reviewer PS and the handling Editor declared their shared affiliation.
